# A scATAC-seq atlas of chromatin accessibility in axolotl brain regions

**DOI:** 10.1038/s41597-023-02533-0

**Published:** 2023-09-14

**Authors:** Weimin Feng, Shuai Liu, Qiuting Deng, Sulei Fu, Yunzhi Yang, Xi Dai, Shuai Wang, Yijin Wang, Yang Liu, Xiumei Lin, Xiangyu Pan, Shijie Hao, Yue Yuan, Ying Gu, Xiuqing Zhang, Hanbo Li, Longqi Liu, Chuanyu Liu, Ji-Feng Fei, Xiaoyu Wei

**Affiliations:** 1 College of Life Sciences, University of Chinese Academy of Sciences, Beijing, 100049 China; 2 BGI-Hangzhou, Hangzhou, 310012 China; 3 BGI-Shenzhen, Shenzhen, 518103 China; 4 BGI College & Henan Institute of Medical and Pharmaceutical Sciences, Zhengzhou University, Zhengzhou, 450000 China; 5 Department of Pathology, Guangdong Provincial People’s Hospital (Guangdong Academy of Medical Sciences), Southern Medical University, Guangzhou, Guangdong 510080 China; 6 Key Laboratory of Brain, Cognition and Education Sciences, Ministry of Education; Institute for Brain Research and Rehabilitation, South China Normal University, Guangzhou, 510631 China; 7 College of Life Sciences, Nankai University, Tianjin, 300071 China; 8 Medical Research Institute, Guangdong Provincial People’s Hospital (Guangdong Academy of Medical Sciences), Southern Medical University, Guangzhou, 510080 China; 9 Guangdong Cardiovsacular Institute, Guangdong Provincial People’s Hospital, Guangdong Academy of Medical Sciences, Guangzhou, 510080 China; 10 BGI-Qingdao, Qingdao, 266555 China; 11 Lars Bolund Institute of Regenerative Medicine, Qingdao-Europe Advanced Institute for Life Sciences, BGI-Qingdao, Qingdao, 266555 China; 12 School of Medicine, South China University of Technology, Guangzhou, Guangdong 510006 China; 13 School of Basic Medical Sciences, Southern Medical University, Guangzhou, Guangdong 510515 China

**Keywords:** Epigenetics and plasticity, Data processing

## Abstract

Axolotl (*Ambystoma mexicanum*) is an excellent model for investigating regeneration, the interaction between regenerative and developmental processes, comparative genomics, and evolution. The brain, which serves as the material basis of consciousness, learning, memory, and behavior, is the most complex and advanced organ in axolotl. The modulation of transcription factors is a crucial aspect in determining the function of diverse regions within the brain. There is, however, no comprehensive understanding of the gene regulatory network of axolotl brain regions. Here, we utilized single-cell ATAC sequencing to generate the chromatin accessibility landscapes of 81,199 cells from the olfactory bulb, telencephalon, diencephalon and mesencephalon, hypothalamus and pituitary, and the rhombencephalon. Based on these data, we identified key transcription factors specific to distinct cell types and compared cell type functions across brain regions. Our results provide a foundation for comprehensive analysis of gene regulatory programs, which are valuable for future studies of axolotl brain development, regeneration, and evolution, as well as on the mechanisms underlying cell-type diversity in vertebrate brains.

## Background & Summary

The axolotl (*Ambystoma mexicanum*) has remarkable regenerative abilities, including the ability to regenerate its limbs, heart, tail, and spinal cord^1–5^, making it as an ideal model for studying vertebrate regeneration, comparative genomics, and evolution. With the development of technology, great progress has been made in axolotl genome and transcriptome research. In brief, recent advances in single-cell and spatial transcriptomics assays have led to profiling the distribution of cell types of the axolotl telencephalon *in situ* during development and regeneration^6^. Furthermore, by using either species-shared differentially expressed genes or transcription factors (TFs), previous axolotl telencephalon studies have shown the conservation between the medial pallium neurons and amniote hippocampal neurons^7^. Interestingly, the axolotl genome has conserved coding regions with the human genome but is ten times its size, with the majority of the expansion in non-coding regions. With a 32-gigabase-pair genome^8^, non-coding regulatory DNA sequences in axolotls may be proposed to play a distinctive role in environmental adaptation. However, a comprehensive understanding of *cis*-regulatory elements in the axolotl genome, especially in complex organs like the brain, has lagged.

Using techniques such as Assay for Transposase-Accessible Chromatin using sequencing (ATAC-seq) and chromatin immunoprecipitation followed by sequencing (ChIP-seq), candidate *cis*-regulatory elements (cCREs) have been mapped to identify and exploit epigenetic features^9,10^. Due to the extreme cellular heterogeneity of the brain, conventional assays that use bulk tissue samples have difficulty identifying cCREs in specific cell types. Single-cell ATAC sequencing (scATAC-seq) is a powerful technique that allows us to identify the regulatory regions of the genome that are active in individual cells^11–14^. It was developed to determine chromatin accessibility in various biological scenarios and the corresponding transcriptional programs. In recent years, scATAC-seq has gained significant attention in the field of neuroscience, because it allows for the investigation of the epigenetic landscape of cell-type-specific transcriptional regulatory sequences in brain regions^15–17^. For example, regulatory elements linked to *Cux2* and *Foxp2*, which are highly expressed only in layers II–IV and layer VI of mice, respectively, are only accessible in cells of the corresponding neuron clusters, reflecting the heterogeneity of chromatin accessibility in different layers of the prefrontal cortex^15^.

In this study, we profiled a comprehensive chromatin accessibility landscape across the axolotl brain with scATAC-seq^16,18,19^, including the olfactory bulb (OB), telencephalon (Tel), diencephalon and mesencephalon (DM), hypothalamus and pituitary (HP), and rhombencephalon (Rho). We were able to obtain single-cell chromatin accessibility for 81,199 cells after applying quality control. By analysing the chromatin accessibility profiles of different cell types, we have identified unique sets of regulatory elements that are specific to each cell type, including microglia, GABAergic, glutamatergic, ependymoglia cell (EGC), and so on. Overall, our findings provide insights into the regulatory mechanisms that underlie the diverse functions of different cell types in axolotl brain regions, and serve as a foundation for future studies aimed at understanding the transcriptional programs that drive the distinguishing features of axolotl brain regions.

## Methods

### Tissue collection

The Biomedical Research Ethics Committee of Guangdong Provincial People’s Hospital (license number: KY-Q-2022-395-01) approved the use of animals in this study. The *d/d* strain of axolotl was used for all experiments without sex bias in the research. Animals were bred and maintained in freshly dechlorinated tap water at 18–20 °C with a 12 h/12 h light-dark cycle in the Laboratory of Neural Development and Regeneration, Guangdong Provincial People’s Hospital. Totally 8 adult axolotls were sacrificed, and OB, Tel, DM, HP, and Rho were harvested in this study. In brief, axolotls were deeply anaesthetized using 0.03% ethyl-p-aminobenzoate. Each brain region from 8 animals was dissected from the axolotl head. Then, each brain region was pooled into a separate tube for single-nucleus isolation, respectively. The samples were snap-frozen in liquid nitrogen and then transferred to a −80 °C freezer for storage before dissociation.

### Nuclear isolation from frozen brain tissue

**Fig. 1:**
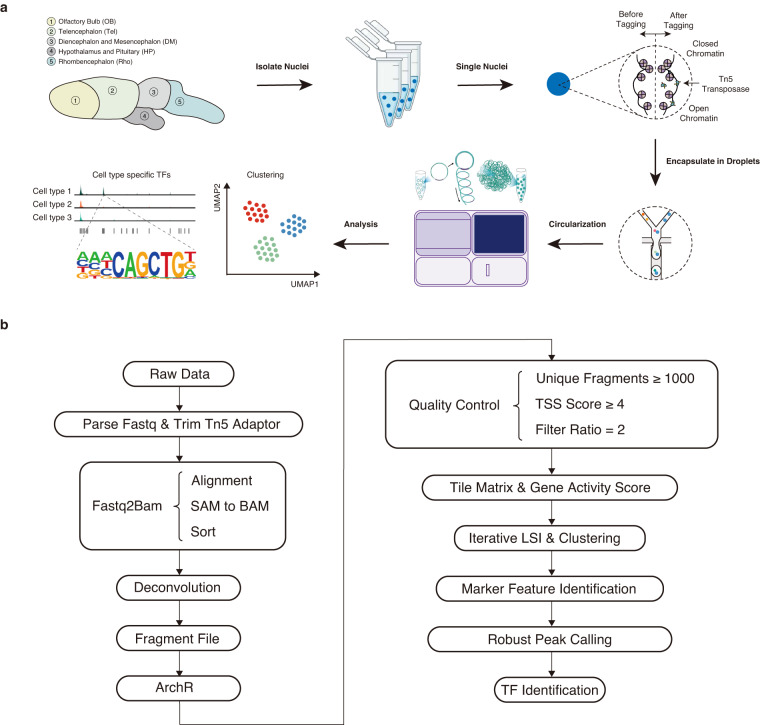
Schematic diagram illustrating the experimental and data analysis processes of scATAC-seq in the axolotl brain. (**a**) Cartoon illustrates the main experimental and analytical processes. Five different regions from the brain of adult axolotl were collected for single-cell Assay for Transposase Accessible Chromatin (scATAC-seq). Region 1, olfactory bulb (OB); region 2, telencephalon (Tel); region 3, diencephalon and mesencephalon (DM); region 4, hypothalamus and pituitary (HP); and region 5, rhombencephalon (Rho). Colors correspond to the five regions. (**b**) Analysis of the scATAC-seq workflow.

The axolotl brain regions focused on this study were the OB, Tel, DM, HP, and Rho (Supplementary Table 1 and Fig. 1a). The single-nucleus preparations were obtained using the Omni-ATAC protocol with certain modifications, as previously described^20^. In brief, nuclei from the frozen brain regions were isolated^21,22^. Tissue was cut into pieces and ground in 2 mL of chilled homogenization buffer [HB; 120 mM Tris pH 7.8 (Sigma), 150 mM KCl (Sigma), 30 mM MgCl_2_ (Invitrogen), 250 mM sucrose (Sigma), 0.1% NP-40 (Roche), 1 × Protease inhibitor cocktail (Roche), 1 mM DTT (Thermo Fisher Scientific), 1% BSA/0.8 × PBS], and incubated on ice for 5 min. The tissue was homogenized by stroking (grinding) to release the nuclei. Then, nuclei were filtered through a 30 μM cell strainer (Sysmex Partec) into a 15 mL centrifuge tube. After centrifugation at 500 g and 4 °C for 5 min, the nuclei were collected and washed twice with 1 mL of chilled blocking buffer [BB, 1% BSA/0.8 × PBS]. After another round of centrifugation, the nuclei were resuspended in 50 μl of 1% BSA/0.8 × PBS and counted by staining with DAPI.

### scATAC-Seq library preparation and sequencing

For the preparation of single-cell ATAC-seq libraries, we employed the DNBelab C Series Single-Cell ATAC Library Prep Set (MGI, #1000021878)^19^. To investigate the transcriptional regulation of different brain regions in axolotl with sufficient and high-quality data, a total of 39 libraries were generated. Specifically, there were 8 libraries for OB, DM, HP, and Rho, and 7 libraries for Tel. After quality control, 25 high-quality libraries were retained, including 6 from the OB, 4 from the Tel, 3 from the DM, 6 from the HP, and 6 from the Rho. The barcoded scATAC-seq libraries were generated from the transposed single-cell suspensions. In summary, the protocol involved droplet encapsulation, pre-amplification, emulsion breakage, collection of capture beads, DNA amplification, and purification. Following this, an indexed sequencing library was prepared as per the user guide. Finally, we used the Qubit ssDNA Assay Kit (Thermo Fisher Scientific) to measure the concentrations of the sequencing libraries. The library was sequenced on the MGI DNBSEQ-T1 platform using the following read lengths: 50 bp for read 1, 70 bp for read 2, and 10 bp for the sample index sequencing scheme of the China National GeneBank^23^ (CNGB).

### scATAC-seq raw data processing

The processing of scATAC-seq data involved the following steps. First, the raw reads were separated into insertions and barcodes and then filtered using PISA (version 1.1) with a minimum sequencing quality of 20; the software is available at https://github.com/shiquan/PISA. The sequencing reports for the scATAC-seq datasets are summarized in Table 1. Next, the filtered reads were aligned to the axolotl genome^8^ using BWA (version 0.7.17-r1188)^24^, and the resulting BAM files were processed using bap2 (version 0.6.2)^25^ to identify barcodes from the same cell (Fig. 1b).

**Table 1:** scATAC-seq metadata and mapping statistics.

Sample ID	Total reads	Reads Pass QC	Estimated number of cells	Mean fragments per cell	TSS Enrichment	Accessible peaks
OB-1	375,820,925	339,441,460	3,651	5,854	20.86	107,713
OB-2	330,716,617	297,016,594	3,594	5,331	20.52	112,426
OB-3	160,832,997	145,264,363	2,922	4,013	19.17	113,486
OB-4	236,547,927	213,626,433	3,243	4,073	17.38	113,332
OB-5	167,666,804	152,023,492	2,793	4,332	18.52	108,282
OB-6	428,341,216	389,533,502	3,441	6,160	20.71	107,890
Tel-1	282,998,946	256,340,446	3,975	5,298	20.98	112,898
Tel-2	170,791,145	154,361,037	3,040	4,385	21.03	114,833
Tel-3	227,647,481	206,999,855	3,376	5,319	20.82	116,451
Tel-4	322,666,500	287,979,852	2,792	6,257	22.21	115,214
DM-1	413,928,279	370,755,560	4,158	5,026	14.44	112,805
DM-2	457,122,784	409,216,317	3,862	5,342	13.62	112,953
DM-3	423,715,798	379,098,525	3,530	6,385	13.85	112,170
HP-1	359,720,792	322,561,635	3,271	6,693	21.59	114,015
HP-2	660,417,261	591,007,407	3,112	8,748	23.20	116,237
HP-3	311,679,700	280,355,891	2,983	6,476	20.93	111,791
HP-4	266,567,225	239,190,771	2,634	5,705	21.74	113,598
HP-5	614,646,512	551,399,386	3,140	8,680	22.72	116,423
HP-6	355,725,790	315,208,623	3,197	5,812	20.71	114,941
Rho-1	604,099,350	539,158,670	2,955	7,493	21.32	114,483
Rho-2	720,833,075	649,038,101	3,338	8,572	20.53	114,006
Rho-3	493,338,543	443,708,686	3,025	8,727	20.87	115,696
Rho-4	427,845,614	386,259,021	2,994	8,268	20.22	115,198
Rho-5	338,051,493	303,840,682	3,190	6,164	19.44	116,404
Rho-6	721,120,639	648,648,015	2,983	8,896	21.75	116,036
Total/Average	9,872,843,413	8,872,034,324	81,199	6,320	19.97	113,571

### Quality control (QC) of the scATAC-seq downstream analysis

ArchR (version 1.0.2)^26^ was used to filter low quality cells with the following criteria: unique fragments (nFrags) ≥ 1000 and transcription start site (TSS) score ≥ 4 of each library. Then, we calculated the doublet score by the ‘addDoubletScores’ function with the parameters of filterRatio = 2 and potential doublets were removed based on the ArchR^26^.

### scATAC-seq gene activity scores

We used ArchR (version 1.0.2)^26^ to calculate gene activity scores. Briefly, considering the 32-gigabase-pair axolotl genome^8^, to determine whether each cell was accessible within each window, we created 100000 bp windows across the genome. ArchR (version 1.0.2)^26^ uses a weighted average of the accessibility of the peaks, where the weight of each peak is determined by its proximity to the transcription start site. We calculated gene activity scores using the ‘addGeneScoreMatrix’ function. Furthermore, due to the sparsity of scATAC-seq data, we employed the MAGIC (Markov Affinity-based Graph Imputation of Cells) imputation technique to impute gene activity scores from bolstering signal strength and reducing noise by sharing information with similar nearby cells.

### Latent semantic indexing (LSI) clustering of scATAC-seq

Analyses of the scATAC-seq data were conducted using ArchR^26^. LSI dimensionality reduction was performed with ‘addIterativeLSI’ function in ArchR^26^. By using Seurat’s ‘FindClusters’ function, we clustered the data with parameters: reducedDims = ‘IterativeLSI’, method = ‘Seurat’, and resolution = 0.8. Markers of each cluster were identified by the ‘getMarkerFeatures’ function^26^ (FDR ≤ 0.05 & Log2FC ≥ 0.25), and cell type annotation was procured using well-known cell-type-specific markers obtained from the MAGIC gene activity score.

### Integration of library

As our data were produced from the same batch, no significant batch effects were observed. The integration of our libraries was performed by simply merging them using ArchR^26^. To accomplish this, we first created a project using ‘ArchRProject’ function. Next, we applied dimensionality reduction to our data using the ‘addIterativeLSI’ function with ‘TileMatrix’ matrix. Subsequently, we incorporated UMAP embedding of reduced dimensions object using ‘addUMAP’ function, allowing us to visualize different brain regions by ‘plotEmbedding’ function. Finally, we calculated the Pearson correlation coefficients between different libraries by ‘cor’ function with the gene activity matrix.

### Peak calling of scATAC-seq

The model-based analysis of ChIP-seq (MACS2)^27^ (https://github.com/hbctraining/Intro-to-ChIPseq) callpeak command was used to perform peak calling on the Tn5-corrected insertions (representing each end of the Tn5-corrected fragments) for each cell type. In brief, the ‘addGroupCoverages’ function was used to create pseudo-bulk replicates for each cell type and the ‘addReproduciblePeakSet’ function (parameters: groupBy = ’Clusters’, pathToMA-CS = pathToMacs2, ‘- nomodel’, genomeSize = 2,782,028,915) was used to call peaks. Next, the ‘getMarkerFeatures’ function was utilized to define the marker peaks for each cell type, and the ‘getMarkers’ function was applied to get the marker peaks.

### Motif enrichment analysis

We annotated motif using the ‘addMotifAnnotations’ function of ArchR with the default parameters: motifSet = ‘cisbp’, name = ‘Motif’, species = ‘homo sapiens’, version = 2. Then, with the marker peaks, motif enrichment was performed by the ‘peakAnnoEnrichment’ function, and the top 18 cell-type-specific motifs were shown in the heatmap.

### Assigning gene to the scATAC-seq peaks

To assign genes to scATAC-seq peaks, it required axolotl genome annotation object and gene annotation object. Firstly, we forged a BSgenome package for the axolotl genome annotation following the official tutorial (https://github.com/Bioconductor/BSgenome). Briefly, we forged axolotl BSgenome package by the ‘forgeBSgenomeDataPkg’ function with the seed file and the published axolotl genome. Then, we created axolotl genome annotation by ‘createGenomeAnnotation’ function (parameters: genome = BSgenome.Axolotl). Secondly, to create gene annotation object, we utilized ‘createGeneAnnotation’ function and 3 GRanges object (GRanges object containing gene coordinates; GRanges object containing gene exon coordinates; GRanges object containing standed transcription start site coordinates for computing TSS enrichment scores downstream) as input. Finally, scATAC-seq peaks were assigned genes by creating an ArchR project from the provided ArrowFiles with ‘ArchRProject’ function (parameters: geneAnnotation = axolotl gene annotation, genomeAnnotation = axolotl genome annotation). With the annotated ArchR project, scATAC-seq peaks were assigned genes by using the peak-to-gene assignment algorithm of ArchR based on their proximity to the transcription start site (TSS) of the nearest gene.

### Gene ontology (GO) enrichment analysis

Gene set enrichment analysis was processed with ‘enrichGO’ function (parameters: OrgDb = org.Hs.eg.db, ont = ‘BP’) of the clusterProfiler package (version 4.4.4). The marker peaks of cell type used as input (FDR ≤ 0.05 & Log2FC ≥ 0.25) were annotated based on proximity to the TSS of the nearest genes.

## Data Records

We presented chromatin accessibility landscapes for different regions of the axolotl brain, providing valuable insights into the epigenetic regulation mechanisms of brain function and cell heterogeneity. Our data set consists of chromatin accessibility landscapes for 81,199 high-quality single-cells from the OB, Tel, DM, HP, and Rho regions of the axolotl brain, including 3–6 libraries of each region. Figure 1 provides an overview of the laboratory bioinformatical and analysis workflow. Table 1 displays the quality of each library. The raw fastq and fragments data have been deposited in the CNGB Nucleotide Sequence Archive (CNP0004118^28^). The raw fastq data have also been submitted to the NCBI Sequence Read Archive (PRJNA990676^29^). Additionally, the peak matrices and metadata has been uploaded to Figshare (10.6084/m9.figshare.22548400.v7)^30^. The Supplementary Table 1 (available at Figshare)^30^ exhibited libraries correlation between the accession IDs in the CNGB or NCBI and the sample IDs.

## Technical Validation

### Quality control of scATAC-seq

In this study, nuclei were extracted from the OB, Tel, DM, HP, and Rho of adult axolotl and were prepared for scATAC-seq (see Methods) (Supplementary Table 1 and Fig. 1a)^30^. The total number of raw reads was 9,872,843,413, with 8,872,034,324 reads passing QC (Table 1). The raw data were processed via the standard pipeline (Fig. 1b).

**Fig. 2:**
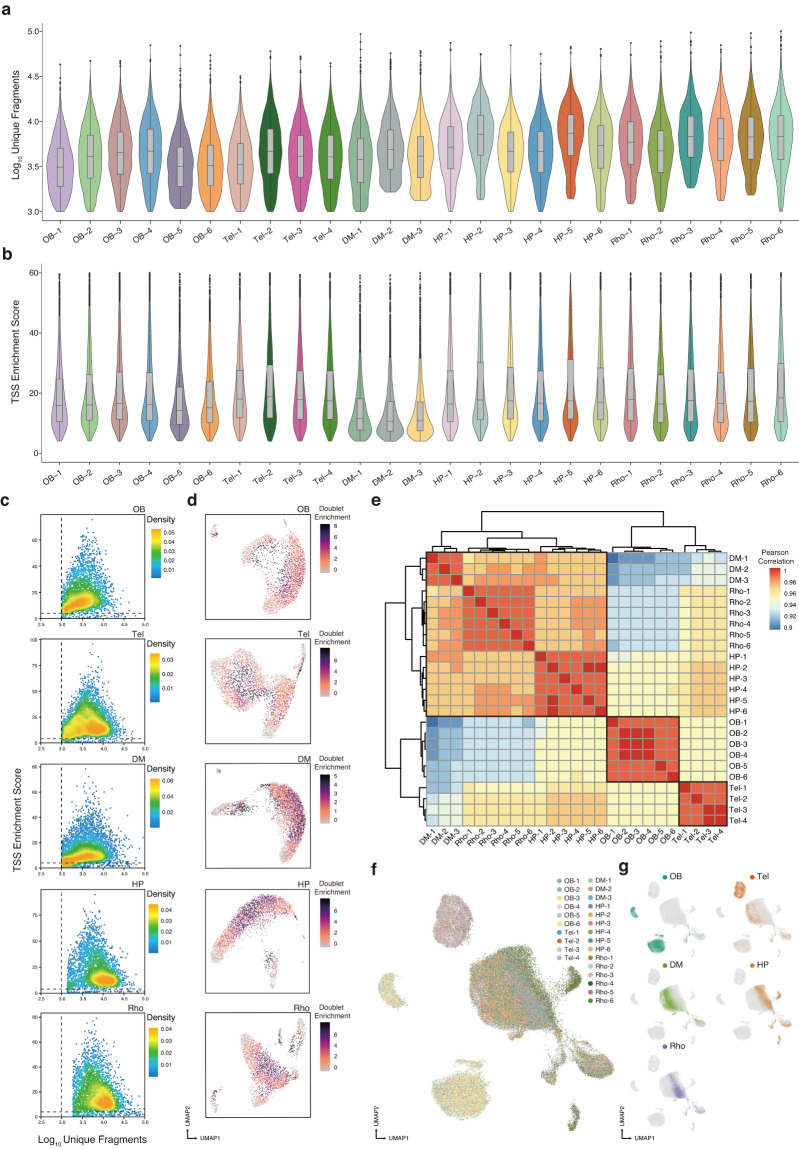
Quality metrics for scATAC-seq. (**a**) Violin plot illustrating the distribution of log_10_ (unique fragments) for each library. (**b**) Violin plot of the transcription start site (TSS) enrichment score for each library. (**c**) Quality control (QC) filtering plots of the TSS enrichment fractions vs unique cell fragments for each cell from the olfactory bulb (OB), telencephalon (Tel), diencephalon and mesencephalon (DM), hypothalamus and pituitary (HP), and rhombencephalon (Rho). (**d**) UMAP of scATAC-seq data showing simulated doublet enrichment over expectation. (**e**) Heatmap clustering of correlation coefficients across 25 libraries of scATAC-seq profiles. (**f**) A UMAP visualization of the 25 libraries, each color of which represents a per library interaction of the five brain regions. (**g**) A UMAP visualization representing the five brain regions; Cells are colored by brain region in the plots.

After quality control, we obtained a satisfying set of single-cell chromatin accessibility profiles for axolotl brain regions (Fig. 2a–c). We eliminated doublets by applying a filterRatio parameter of 2 in ArchR^26^ (Fig. 2d), and subsequently examined the chromatin landscape of the 81,199 single cells to investigate cell-type-specific regulatory elements. The average unique fragments of cells remaining after quality filtering was 6,281; the average of TSS enrichment was 19.97, the average accessible peaks of each library were 113,571 (Table 1). To identify reproduced peaks between technical replicates, we computed Pearson correlation coefficients to assess the similarity between technical replicates across regions, based on gene activity (Fig. 2e). UMAP showed the heterogeneity of gene activity in different brain regions, which is probably indicative of differential functioning (Fig. 2f,g). For example, the Tel serves as the hub for sensory processing, oversees voluntary movements and activities, and is linked to sophisticated cognitive skills such as emotions, learning, and memory^31^, whereas the primary role of the HP is to manage neuroendocrine processes, with the paraventricular region housing cell bodies that project to the hypophysis and secrete hormones^32^.

### Identification of accessible cell-type chromatin and comparison of regions

**Fig. 3:**
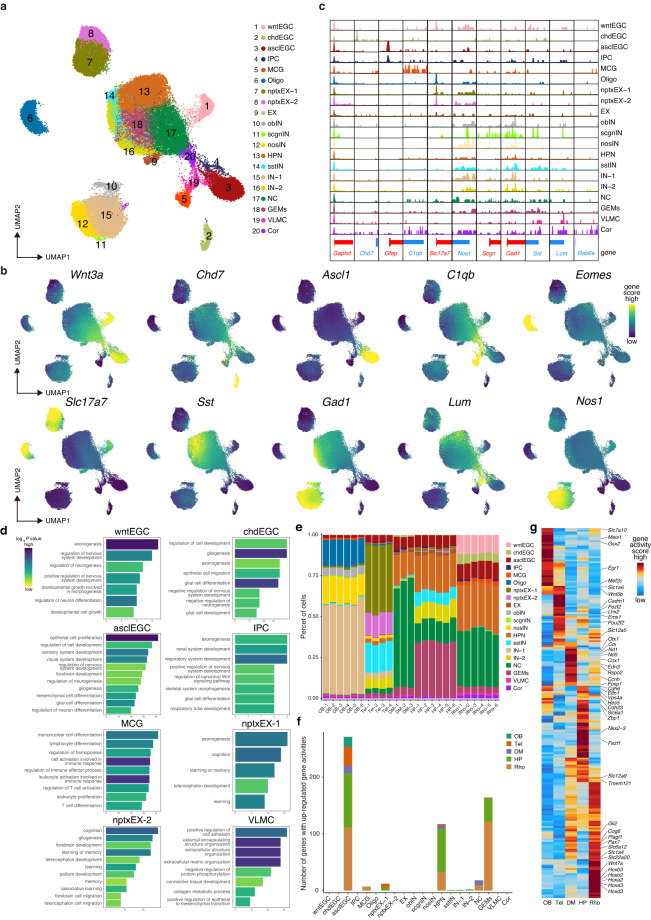
Single-cell chromatin accessibility and diversity between assessed regions of the axolotl brain. (**a**) UMAP of 20 clusters as identified by scATAC-seq. Cells coloration indicates annotation. (**b**) UMAP visualization of cell-type-specific gene activity score. (**c**) Genome browser view of the aggregated scATAC-seq chromatin accessibility profiles of the housekeeping gene (*Gapdh*) and the cell-type-specific gene loci. (**d**) Bar plot exhibiting the representative GO enrichment pathways of cell types. (**e**) Histogram displaying the distribution of cell types across various brain regions in different libraries. (**f**) Histogram of the number of up-regulated genes of the same cell type in different axolotl brain regions. (**g**) Heatmap of asclEGC-specific gene activity scores in the five brain regions.

We generated scATAC-seq data across the axolotl OB, Tel, DM, HP, and Rho brain regions. Projecting cells into a low-dimensional embedding (optimized iterative LSI) and estimating gene expression by aggregating the accessibility across the regulatory region of the gene^26^ (gene activity score), we identified 20 cell types (Fig. 3a). In brief, we identified three homeostatic EGCs, named asclEGC (*Ascl1*^+^*, Gfap*^+^*, Krt18*^+^*, Sfrp1*^+^), chdEGC^33^ (*Chd7*^+^, *Olig1*^+^, *Sox10*^+^, *S100a10*^+^) and wntEGC (*Nrep*^+^, *Wnt3a*^+^). We also detected GABAergic (*Gad1*^+^*, Gad2*^+^), glutamatergic (*Slc17a*^+^) neurons, with GABAergic neurons subtypes of obIN (*Foxp2*^+^), scgnIN (*Scgn*^+^), nosIN^34,35^ (*Nos1*^+^), sstIN (*Sst*^+^), and glutamatergic subtypes, including nptxEX-1 (*Nptx1*^+^,*Tbr1*^+^) and nptxEX-2 (*Nptx2*^+^)^6,7^. We also observed HP neuron^36,37^ (HPN, *Nefm*^+^, *Nova1*^+^) in our data. The brain-resident non-neuronal cell-types microglia (MCG, *C1qb*^+^, *Cd74*^+^), intermediate progenitor cell (IPC, *Olig1*^+^), oligodendrocytes (Oligo, *Eomes*^+^*, Olig2*^+^), vascular leptomeningeal cell (VLMC, *Lum*^+^*, Dcn*^+^), neuronal ciliary^38,39^ (NC, *Ssx2ip*^+^, *Ccdc66*^+^) and two secreting cell types of GEMs (possibly neurosecretory oxytocin or vasopressin neuron terminals^40^, *Nkx2-1*^+^, *Nr5a1*^+^) and corticotropic cells (Cor, *Adh1*^+^, *Rab6a*^+^, *Tll2*^+^) were distinguished by their markers (Fig. 3b, Supplementary Fig. 1, Supplementary Tables 2, 3, Supplementary Tables available at Figshare)^30^. To better understand the functions of these cell types, we performed GO analysis on the differential peaks specific to each cell type. The results of the GO analysis revealed a significant correlation between the identified cell types and their corresponding GO pathways. For example, asclEGC was enriched in the function of differentiation or proliferation pathways, while MGC was enriched in immune-related functions, such as the GO terms of cell activation involved in immune response and so on (Fig. 3d).

Chromatin accessibility analysis was used to identify the differential accessibility regions (DARs) of those 20 cell types (Fig. 3c). For example, peaks were identified specifically within the TSS region of the *Gfap* marker genes that were accessible in both asclEGC and IPC. In addition, signals around *Chd7* were specifically enriched in the chdEGC region but not in other cell types. These phenomena strongly suggest that our data are of adequate quality. To identify responding cells across regions, we investigated the varied cell-type compositions of 25 libraries by percent constituency (Fig. 3e). Interestingly, asclEGC increased gradually from the anterior to the posterior axis, and wntEGC and chdEGC were specifically enriched in Rho. Next, the diversity of cellular compositions across different regions of the axolotl brain was analysed in detail and summarized in Fig. 3f. We found asclEGC, HPN, and GEMs have more distinct gene regulation in regions with a large number of different up-regulated gene activities. As *Gfap* is a stem-cell associated protein that, like stem cells, exhibits a similar response to injury^31,32,41,42^, we analysed asclEGC (with *Gfap* chromatin accessible) and found *Egr1* and *Meis1* are accessible in OB; *Mef2c*, *Slc1a6*, *Wnt5b*, *Lhx2* in Tel; Col and the Nd family in DM; *Chd6*, *Nkx2-3* in HP; *Pax7* and the Hox family in Rho, respectively (Fig. 3g). Therefore, this analysis strongly suggests that the scATAC-seq profiles of axolotl brain regions can effectively and accurately identify the accessible chromatin regions within the axolotl genome and provides a direct theoretical basis reference that can be exploited by future studies.

### Inferring cell type-specific transcription factors

**Fig. 4:**
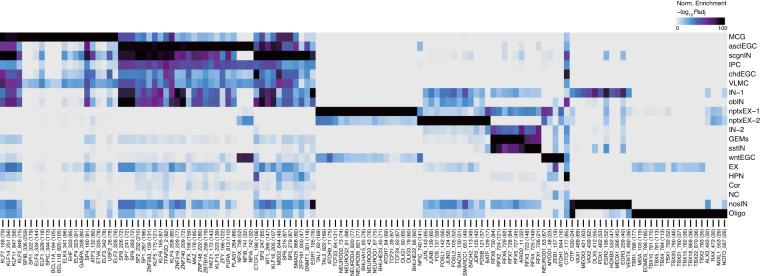
Identification of cell-type-specific chromatin transcription factors (TFs). Heat map clusters indicate cell-type-specific TFs.

To gain a better understanding of the regulatory mechanisms governing the chromatin landscape, we implemented peak calling to generate a set of 121,697 cell-type differential peaks based on pseudobulk chromatin accessibility. Then, we used ArchR^26^ to identify transcription factors that exhibit strong correlation with cell-type-specific open chromatin and compared these results with previous studies (Fig. 4). Conformably, the master regulators have been shown to be closely related to specific functions of cell types. For example, MCG is enriched for motifs of the BCL11 family (BCL11A^43^, BCL11B^44^). This was supported by the previous study of BCL11A deletion causes apoptosis in immature B cells and common lymphoid progenitors, and also results in delayed or deficient lymphoid development of hematopoietic stem cells to B, T, and NK cells in adult mice^43^. Besides, BCL11B prevents autoimmune disorders by controlling multiple regulatory T cell gene expression programs^44^. EGCs are enriched for motifs that distinguish progenitor cells from other cell types, in the agreement of the characterization of proliferation in EGCs^31,32,41,42^. In brief, we observed strong enrichment of PLAG1 in asclEGC, which is consistent with regulation of progenitor cell proliferation and neurogenesis during telencephalic development of mice^45^. We also found NEUROD1 is enriched in wntEGC in our dataset. NEUROD1 promotes transit-amplifying progenitors that contribute to the generation of the majority of the excitatory neurons of the neuroepithelium of the dorsal telencephalon in early development^46–48^. Moreover, chdEGC is enriched with CTCF, which regulates functional neural development and neuronal diversity^49^. Other well-known cell-type-specific motifs such as the Oligo (TBR1^50^, EOMES^51^), nptxEX-1 (NEUROG2, NEUROD2, NEUROD4, NEUROD6^52^, BHLHE22), nptxEX-2 (BACH1, BACH2, the JUN^53^, NFIC, and FOSL families), nosIN (DLX family), and GEMs (RFX family) were enriched in our data. Taken together, cell-type-specific TFs that have been reported in previous studies are also present in our data, exhibiting similar patterns of enrichment, which indicates the accuracy of our cell type identification and the high-quality of our data. Therefore, we provide a resource-rich and high-quality data of chromatin landscape of the axolotl brain.

## Usage Notes

The scATAC-seq data processing pipeline, including read mapping and peak calling, were run on the Linux operating system. All R source code used for downstream data analyses and visualization are provided online (10.6084/m9.figshare.22548400.v7)^30^.

### Supplementary information

Supplementary Figure

Supplementary Table 1

Supplementary Table 2

Supplementary Table 3

## Data Availability

The R code used to identify cell subclusters and profile cell type-specific chromatin accessible regions of the axolotl brain are available online (10.6084/m9.figshare.22548400.v7)^30^.
