# Supplementary material for: A scATAC-seq atlas of chromatin accessibility in axolotl brain regions
Source: Sci Data. 2023 Sep 14;10:627. doi: 10.1038/s41597-023-02533-0 (PMC10502032; doi:10.1038/s41597-023-02533-0)
Supplement: Supplementary file 1 — Supplementary Figure [file 41597_2023_2533_MOESM1_ESM.pdf]

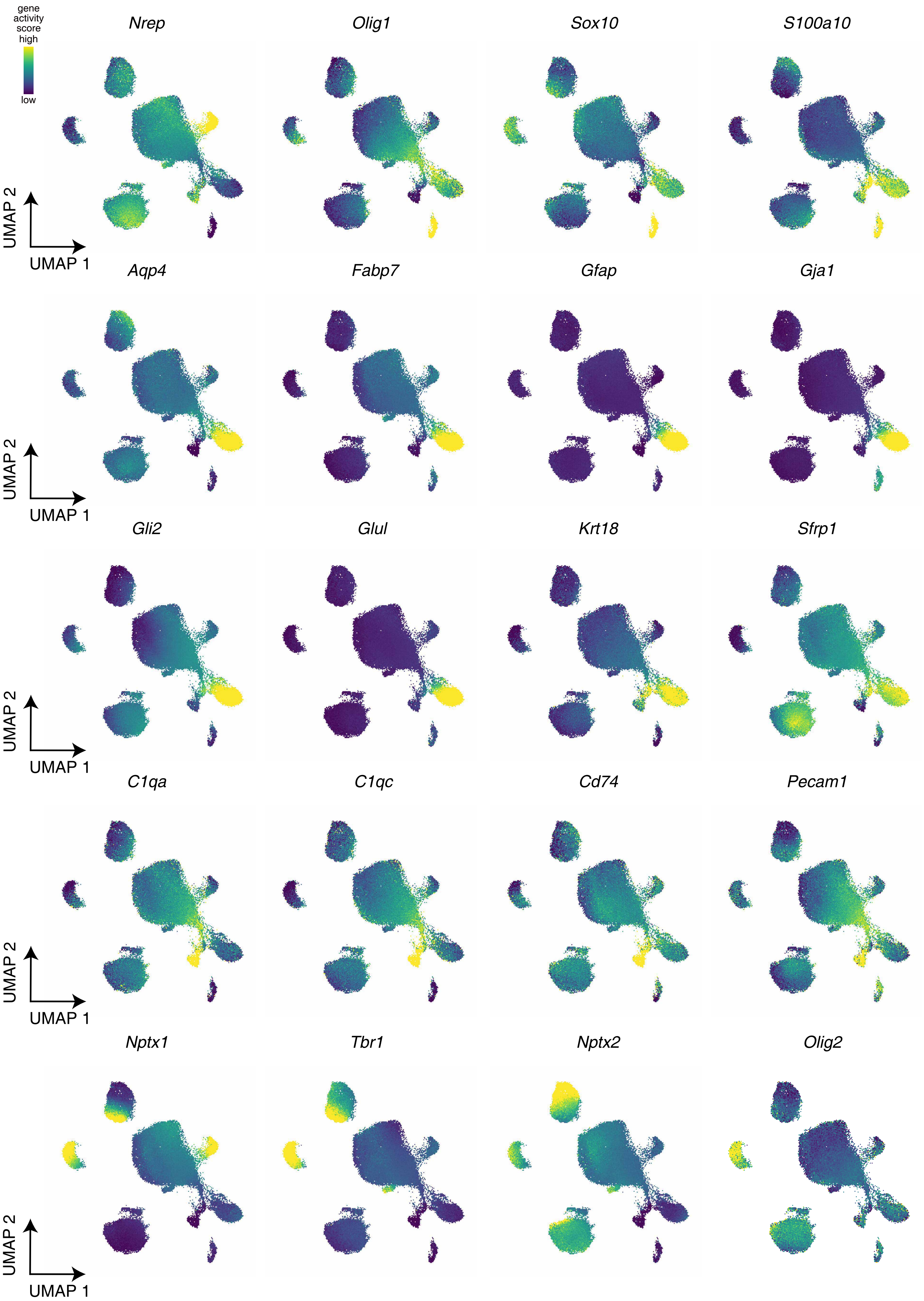
gene activity score
high
low
Nrep
Olig1
Sox10
S100a10
Aqp4
Fabp7
Gfap
Gja1
Gli2
Glul
Krt18
Sfrp1
C1qa
C1qc
Cd74
Pecam1
Nptx1
Tbr1
Nptx2
Olig2
UMAP 1
UMAP 2

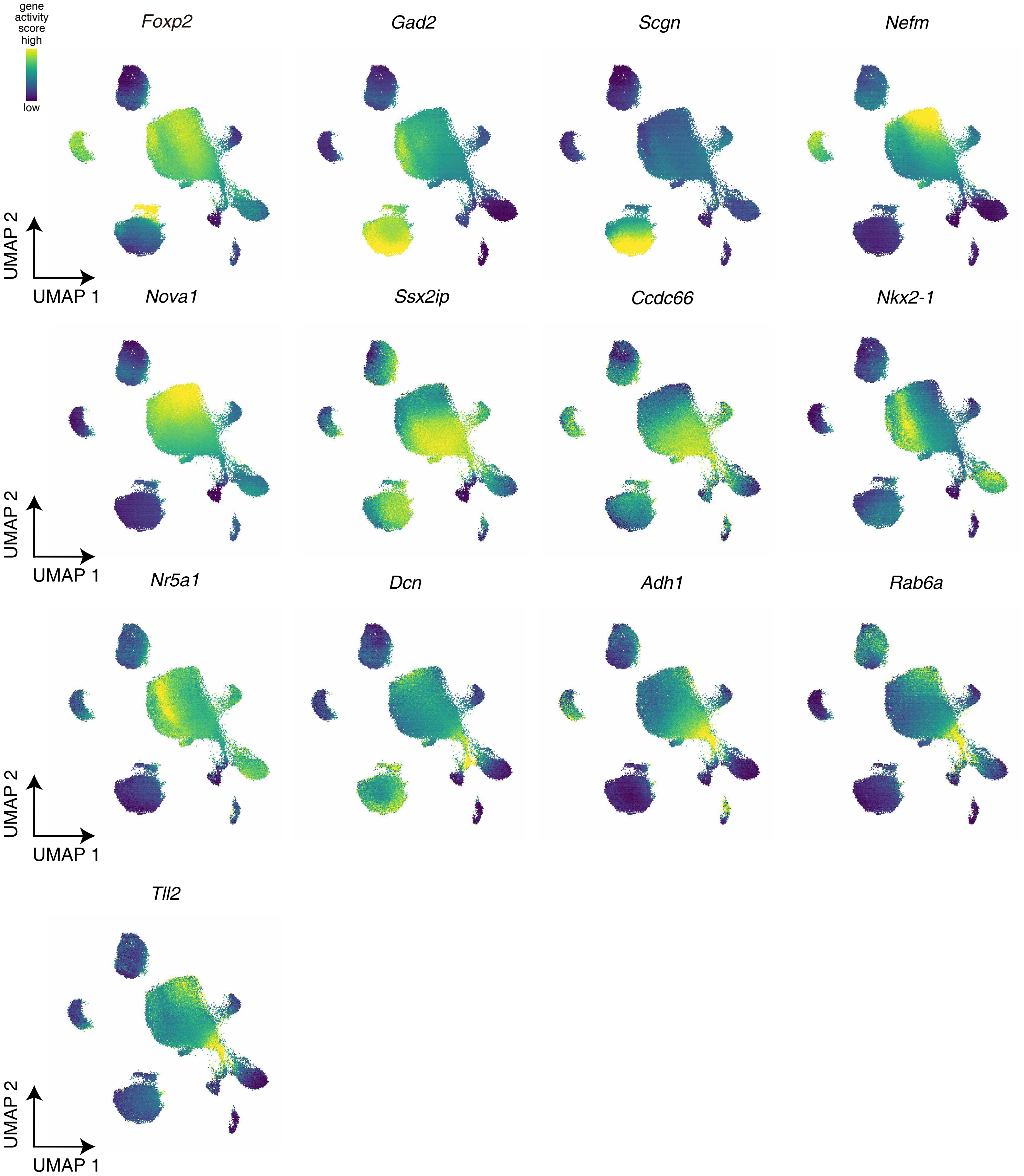
gene activity score
high
low
Foxp2
Gad2
Scgn
Nefm
Nova1
Ssx2ip
Ccdc66
Nkx2-1
Nr5a1
Dcn
Adh1
Rab6a
Tll2
UMAP 2
UMAP 1

Supplementary Figure 1. Identification of cell-type-specific marker genes.
The UMAP visualization of gene activity score of cell-type-specific markers, corresponding to Fig. 3b.
