# Supplementary material for: A scATAC-seq atlas of chromatin accessibility in axolotl brain regions
Source: Sci Data. 2023 Sep 14;10:627. doi: 10.1038/s41597-023-02533-0 (PMC10502032; doi:10.1038/s41597-023-02533-0)
Supplement: Supplementary file 3 — Supplementary Table 2 [file 41597_2023_2533_MOESM3_ESM.docx]

| Cluster | Cell Type | Gene marker |
| --- | --- | --- |
| 1 | wntEGC | *Nrep, Wnt3a* |
| 2 | chdEGC | *Chd7, Olig1, Sox10, S100a10* |
| 3 | asclEGC | *Ascl1, Aqp4, Fabp7, Gfap, Gja1, Gli2, Glul, Krt18, Sfrp1* |
| 4 | IPC | *Olig1* |
| 5 | MCG | *C1qa, C1qb, C1qc, Cd74, Pecam1* |
| 6 | Oligo | *Eomes, Olig2* |
| 7 | nptxEX-1 | *Nptx1, Tbr1, Slc17a7* |
| 8 | nptxEX-2 | *Nptx2, Slc17a7* |
| 9 | EX | *Slc17a7, Tbr1* |
| 10 | obIN | *Foxp2, Gad1, Gad2* |
| 11 | scgnIN | *Scgn, Gad1, Gad2* |
| 12 | nosIN | *Nos1, Gad1, Gad2* |
| 13 | HPN | *Nefm**,* *Nova1* |
| 14 | sstIN | *Sst, Gad1, Gad2* |
| 15 | IN-1 | *Gad1, Gad2* |
| 16 | IN-2 | *Gad1, Gad2* |
| 17 | NC | *Ssx2ip, Ccdc66* |
| 18 | GEMs | *Nkx2-1, Nr5a1* |
| 19 | VLMC | *Lum, Dcn* |
| 20 | Cor | *Adh1, Rab6a, Tll2* |

Supplementary Table 2. Cell type-specific marker gene list.
